# Impact of the Incorporation of Nano-Sized Cellulose Formate on the End Quality of Polylactic Acid Composite Film

**DOI:** 10.3390/nano12010001

**Published:** 2021-12-21

**Authors:** Yidong Zhang, Chao Liu, Meiyan Wu, Zhenqiu Li, Bin Li

**Affiliations:** 1 CAS Key Laboratory of Biofuels, Qingdao Institute of Bioenergy and Bioprocess Technology, Chinese Academy of Sciences, Qingdao 266101, China; zhangyidongqust@163.com (Y.Z.); liuchao@qibebt.ac.cn (C.L.); wumy@qibebt.ac.cn (M.W.); 2 College of Marine Science and Biological Engineering, Qingdao University of Science and Technology, Qingdao 266011, China; lizhenqiu@qust.edu.cn

**Keywords:** polylactic acid composite films, cellulose nanofibril, cellulose nanocrystal, cellulose formate, sustainable packaging

## Abstract

Polylactic acid (PLA) films with good sustainable and biodegradable properties have been increasingly explored recently, while the poor mechanical property of PLA limits its further application. Herein, three kinds of nano-sized cellulose formate (NCF: cellulose nanofibril (CNF), cellulose nanocrystal (CNC), and regenerated cellulose formate (CF)) with different properties were fabricated via a one-step formic acid (FA) hydrolysis of tobacco stalk, and the influence of the properties of NCF with different morphologies, crystallinity index (*CrI*), and degree of substitution (*DS*) on the end quality of PLA composite film was systematically compared. Results showed that the PLA/CNC film showed the highest increase (106%) of tensile strength compared to the CNF- and CF-based films, which was induced by the rod-like CNC with higher *CrI*. PLA/CF film showed the largest increase (50%) of elongation at the break and more even surface, which was due to the stronger interfacial interaction between PLA and the CF with higher *DS*. Moreover, the degradation property of PLA/CNF film was better than that of other composite films. This fundamental study was very beneficial for the development of high-quality, sustainable packaging as an alternative to petroleum-based products.

## 1. Introduction

The widely used petroleum-based polymers have caused increasing environmental concerns in the world because they have already resulted in the serious contamination of soil and groundwater or the release of hazardous substances into the atmosphere during disposal [1]. Therefore, some biodegradable polymers (such as polylactic acid (PLA), polyhydroxy alkanoates (PHA), and polyhydroxy butyrate (PHB)) have been explored to settle this issue. Among these polymers, PLA derived from renewable crops has been extensively studied for several decades due to its environmental sustainability and biodegradability [2]. Compared with petroleum-based polymers, PLA requires 25–55% less energy to produce [3]. Moreover, the most attractive aspect of PLA, especially with respect to packaging applications, is its non-toxic release during the degradation period. However, despite its advantages, PLA also suffers from series of drawbacks, such as brittleness, poor moisture barrier property, and high migration levels [4,5]. Apart from the abovementioned drawbacks, the low crystallization rate and slow degradation rate of PLA have also limited its further application in some cases [6,7,8]. As known, developing polymer composites by introducing nanofillers was an effective method to overcome these drawbacks. Recently, various types of inorganic nanoparticles with varying geometries and functionalities have been blended and incorporated within PLA, including carbon nanotube [9], hydroxyapatite [10], graphene [11], silver nanoparticles [12], and halloysite nanotubes [13]. However, these nanoparticles are non-biodegradable and not sustainable. 

Compared to inorganic reinforcing nanofillers, nanocellulose (NC, mainly including the fiber-like cellulose nanofibrils (CNF) and the rod-like cellulose nanocrystals (CNC)) has been recognized as one of the optimal reinforcements to develop polymer composites due to its low density, good biocompatibility, and good degradability and renewability [14,15]. Different from the normal-sized cellulose fibers, NC has a unique nano size, high aspect ratio, impressive mechanical properties, and special optical properties [15]. It was reported that a membrane was manufactured from polyvinylidene fluoride nanofiber and CNC for both energy harvesting and oil/water separation application [16]. The good interfacial interaction and good dispersion of nanofillers in the polymer matrix (such as in PLA) are highly needed to achieve the ideal strengthening effect of the composites [17]. It was reported that some compatibilizers (for instance, polyethylene glycol) could improve the dispersion of fillers in composites [18], but they are derived from petroleum-based polymers, which are not renewable. On the other hand, partial acetylation of cellulose could be an effective way to enhance the reinforcing effectiveness of NC in PLA matrix [19]. Similarly, cellulose formate (CF) can be obtained via the esterification between formic acid (FA) and cellulose [20]. The solubility of CF is highly related to its DS, and the properties of CF play an important role in its end application [21,22]. However, most literature only focused on the reinforcing effect of NC on mechanical properties, while the comparison of the properties of NC with different morphology, *CrI,* and *DS* on the quality of resultant PLA composite films was not systematically investigated. 

In our previous work, three types of nano-sized cellulose formate (NCF) products (i.e., CNF, CNC, and regenerated cellulose formate (CF) with distinct characteristics (not only the morphology but also the distinct crystalline structure and DS) were produced by one-pot formic acid (FA) hydrolysis of bleached wood pulp [23]. Among the NCF products, CNF and CNC with *DS* less than 1 were insoluble in FA, but they could be well dispersed in *N*,*N*-dimethylacetamide (DMAc) and dimethyl sulfoxide (DMSO), and they could even be well dispersed in water after high-pressure homogenization [24]. Moreover, the regenerated CF with high *DS* (>1.3) and cellulose II crystalline structure is a new kind of NC, which was regenerated from FA solution by adding water [20]. It is speculated that the CF with the high *DS* could be used as a new kind of nanofiller to reinforce PLA.

Therefore, in this work, the CNF, CNC, and regenerated CF with formate groups were prepared using the FA hydrolysis of tobacco stalk (an agroforestry residue), and the impacts of different properties of the as-fabricated NCF products (particularly for the newly developed regenerated CF) on the quality of the end PLA composite films were comprehensively investigated and compared, including morphology, mechanical properties, nonisothermal crystallization behavior, thermal stability, and degradation property of the final PLA composite films. Thus, this study is beneficial for the development of high-quality, sustainable packaging as a potential alternative to petroleum-based products. 

## 2. Materials and Methods

### 2.1. Materials

Raw tobacco stalk (TS, cellulose 40.5 ± 0.6%; hemicellulose 16.5 ± 0.1%; lignin 24.3 ± 0.2%) was peeled to eliminate roots and barks, and then smashed under 20 mesh for further pretreatment. PLA was supplied by Bright China Industrial Co., Ltd., Shenzhen, China. Ammonium sulfite (NH_4_SO_3_, 90%) was purchased from MACKLIN (Shanghai, China). Urea (CH_4_N_2_O, 99%) was purchased from Beijing Solarbio Science & Technology Co., Ltd., Beijing, China. All other chemicals used were purchased from Sinopharm Chemical Reagent CO., Ltd., Beijing, China, including FA (88 wt%), sodium hydroxide (NaOH, 96 wt%), hydrogen peroxide (H_2_O_2_, 30 wt%), *N*,*N*-dimethylacetamide (DMAc, AR), and other reagents.

### 2.2. Ammonium Sulfite Pretreatment and Bleaching of TS

Ammonium sulfite (AS) pretreatment was carried out in a rotating reactor system (VRD-42SD-A, China National Pulp and Paper Research institute, Ltd., Beijing, China) at 160 °C and 1 MPa for 2 h. The TS powder (50 g) was treated with 20 wt% of AS and 6 wt% of urea (based on the oven-dried mass of TS) to maintain the pretreatment conditions. The liquid-to-solid weight ratio of solvent (water) to TS powder was 6. The as-prepared TS samples were washed with deionized water until pH reached neutral to remove dissolved lignin and residual chemicals. Then, 5 wt.% H_2_O_2_ (based on the oven-dried mass of TS sample) was used to bleach the TS samples at 90 °C for 2 h. The liquid-to-solid weight ratio was 10, and 1 M NaOH solution was used to adjust the pH of the mixture to 10.5. This bleaching process was repeated three times until the snowy-white, bleached sample (referred to as BT) was obtained. Finally, the BT samples were washed with deionized water until pH reached neutral to remove residual chemicals.

### 2.3. Preparation of NCF

The process for the preparation of NCF (i.e., CNF, CNC, and regenerated CF) with FA hydrolysis was performed using the protocol reported previously with small modification [24,25]. Briefly, BT samples were dried at 75 °C for 4 h to remove moisture. Then, BT samples were treated with FA (1: 30, w/v) in an oil bath with 300 rpm mechanical stirring at 95 °C for 6 h. After that, the samples were immediately centrifuged at 10,000 rpm (centrifugal force was 1096× *g*) for 4 min to separate the hydrolysate and precipitate. The hydrolysate was collected for the production of regenerated CF (the CF with a relatively higher *DS* (>1.3) could be well dissolved in FA) [23]. Then, the FA hydrolysate was added in water to regenerate CF and the regenerated CF could be visually observed in water. After that, the regenerated CF could be obtained by vacuum filtration to separate the solid regenerated CF and water. The precipitate was rinsed with deionized water to remove impurities. After that, the precipitate was dispersed in DMAc and subsequently centrifuged at 10,000 rpm (centrifugal force was 1096× *g*) for 5 min. After centrifugal separation, the CNC could be obtained in the supernatant. Then, the sediment was re-dispersed in DMAc with a concentration of 0.2 wt% and then passed through an ATH-BASIC homogenizer (ATH Engineering, Ltd., Beijing, China) three times at 200 bars and 10 times at 600 bars to obtain CNF.

### 2.4. Preparation of the PLA Film and Composite Films

**Figure 1:**
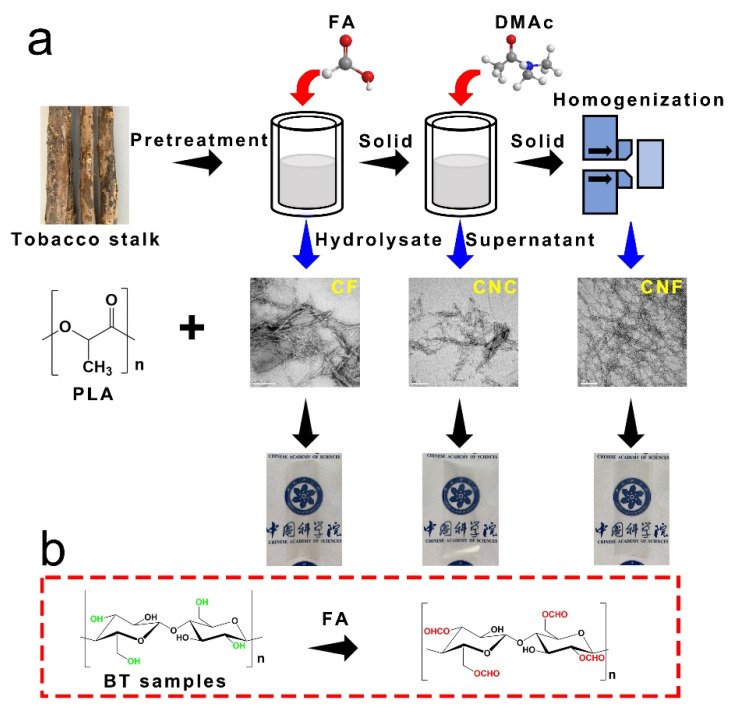
Schematic illustration of the preparation for PLA composite films (**a**) and the reaction between cellulose and FA (**b**).

PLA/CNF, PLA/CNC, and PLA/CF composite films were prepared by a solution casting technique. Firstly, 0.5 g PLA was dissolved in DMAc and stirred at 90 °C for 4 h to make sure that the PLA was well dissolved and the final concentration of PLA was 15 wt%. Then, the certain quantities of CNF, CNC, or regenerated CF (varied from 0.5 to 20 wt% based on the weight of PLA) were added into DMAc solution under ultrasonic mixing for 30 min. Finally, PLA/CNF, PLA/CNC, and PLA/CF composite films (named with the content of NCF) with a thickness of approximately 0.07−0.08 mm were obtained on a glass petri dish via evaporating DMAc. After drying at 110 °C for 3 h, the composite films were obtained. The neat PLA film was prepared by the same process as described above for comparison. The whole process is shown in Figure 1.

### 2.5. Characterization 

Fourier transform infrared (FTIR) analyses were carried out on a Thermo Nicolet FTIR spectrometer (Thermo Nicolet 6700, New York, NY, USA) in the wavenumber range of 400–4000 cm^−1^ with a resolution of 4 cm^−1^. The weight ratio of KBr to NCF sample was 100:1. The crystallinity index (*CrI*) of NCF sample was measured using an X-ray diffractometer (XRD, Bruker Discover D8, Madison, WI, USA). The scattering angle (2*θ*) ranged from 5° to 60° with a scan rate of 0.01°/s. Then, the *CrI* of NCF samples was calculated according to the Segal method [26]. 

The scanning electron microscope (SEM, Hitachi S-4800, Tokyo, Japan) and atomic force microscope (AFM, Agilent 5400, Santa Clara, CA, USA) were employed to observe the morphologies of the film samples. The morphologies of CNF, CNC, and regenerated CF were observed using a Transmission electron microscope (TEM, Hitachi H-7600, Tokyo, Japan). All the images were taken at 100-kV accelerating voltage. Before the TEM test, one drop of NC suspension with a concentration of 0.01 wt% was dropped on a carbon-supported copper grid. After drying, the specimen was dyed with 2 wt% uranyl acetates for 2 h. The dimensions of NC were statistically analyzed by measuring 200 individual nanofibers with clearly identifiable ends (Nano Measurer). 

The mechanical property test of film samples was performed on a CMT6503 universal testing machine (MTS SYSTEMS, Shanghai, China) with a stretching rate of 2 mm/min. The thermal stability was evaluated using a thermogravimetric analyzer (TGA, NETZSCH STA449F5 jupiter, Berlin, Germany). Each sample was heated from 30 to 600 °C at a heating rate of 10 °C min^−1^ under nitrogen (25 mL min^−1^) protection. The onset degradation temperature (*T_o_*) and the maximum degradation temperature (*T_max_*) were obtained by tangent method from TG curves. Water contact angles of composite films were tested using a contact angle goniometer (KINO, SL200B, New York, NY, USA) at ambient temperature. Other methods of characterization are presented in Supporting Information, including *DS*, surface free energy, thermal stability, nonisothermal crystallization behavior, crystallization kinetics, overall migration, and degradation study.

## 3. Results and Discussion

### 3.1. Morphologies and Structure Characteristics 

**Figure 2:**
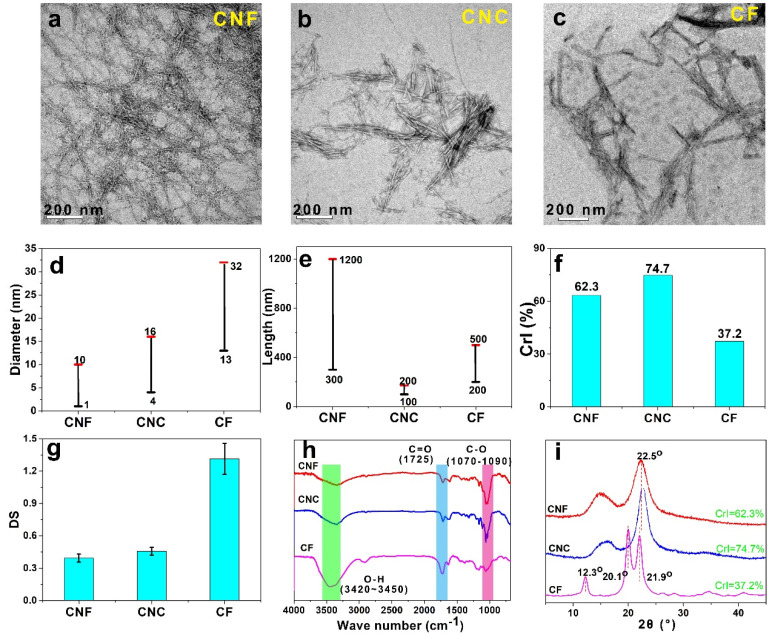
TEM images of CNF (**a**), CNC (**b**), and regenerated CF (**c**); Diameter (**d**), Length (**e**), CrI (**f**), DS (**g**), FTIR spectra (**h**), and XRD patterns (**i**) of CNF, CNC, and regenerated CF.

As mentioned above, the morphologies, diameter, length, *CrI*, and *DS* of three NCF samples were clearly different. TEM images of NCF samples are presented in Figure 2a–c, and the diameter of CNF, CNC, and regenerated CF were 1–10 nm, 4–16 nm, and 13–32 nm, respectively (Figure 2d). As shown in Figure 2e, the length of CNF, CNC, and regenerated CF were 300–1200 nm, 100–200 nm, and 200–500 nm, respectively. Thus, the aspect ratio of CNF, CNC, and regenerated CF were 120–300, 12.5–25, and 15–18, accordingly. The CNC had a rod-like structure because of the removal of the amorphous cellulose during preparation, while the CNF with much larger lengths exhibited a complex and highly entangled network structure. Moreover, the regenerated CF samples showed a large diameter, which was probably due to the low *CrI* caused by dissolution and regeneration. The *CrI* and *DS* of the above NCF samples were further investigated. Compared to the regenerated CF (*CrI*~37.2%), CNF and CNC had higher *CrI* values (62.3% and 74.7%, respectively), which were probably due to the removal of the disordered regions of cellulose and hemicellulose during the pretreatment (Figure 2f). As known, under the reaction conditions of heating and stirring, FA hydrolysis was beneficial to remove amorphous regions of cellulose and hemicellulose [24]. The *DS* values of NCF samples were determined and are presented in Figure 2g. The higher *DS* value (>1.3) of regenerated CF was due to the higher reaction extent between FA and cellulose, thus generating more ester groups and making CF soluble in FA [23]. Thus, three kinds of NCF with different morphologies, diameter, *CrI*, and *DS* were fabricated. 

Furthermore, the FTIR spectra and XRD patterns of NCF samples were recorded to analyze the structure characteristics. Figure 2h shows the FTIR spectra of NCF samples. Similar bands at 3420 cm^−1^ to 3450 cm^−1^ were assigned to O-H stretching vibrations [27]. The bands at 1070 cm^−1^ to 1090 cm^−1^ were assigned to C-O stretching vibrations [10,22,28]. The new band at 1725 cm^−1^ was due to the C=O stretching of ester groups on NCF surface. It was reported that FA could react with hydroxyls of cellulose, generating ester groups [29]. The XRD diffraction patterns of NCF samples are shown in Figure 2i. According to three characteristic peaks (14.8°, 16.5°, 22.5°), CNF and CNC showed the typical cellulose I structure [30]. The diffraction image of regenerated CF exhibited a clear change and revealed the formation of cellulose II structure with a low crystallinity of 37.2%, which was due to the dissolution and regeneration process [31,32]. Cellulose with more amorphous regions had higher reactivity with FA, generating CF with a higher *DS* and lower *CrI*, which was soluble in FA. After regeneration of CF in water, the regenerated CF was re-assembled to cellulose II structure.

### 3.2. Mechanical Property and Model Analysis of PLA Composite Films

**Figure 3:**
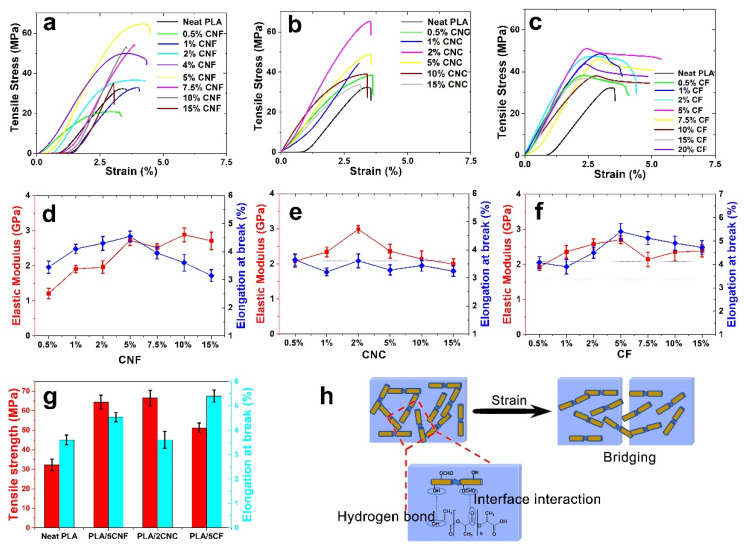
Tensile stress–strain curves of neat PLA film and PLA composite films with different filler contents ((**a**), CNF; (**b**), CNC; (**c**), regenerated CF); The Elastic modulus (*E*) and Elongation at break (*ε*) of neat PLA film and PLA composite films ((**d**), CNF; (**e**), CNC; (**f**), regenerated CF); Mechanical properties of neat PLA film and PLA composite films at the appropriate NCF content (**g**); Schematic illustration for the dispersion and fractured mechanism of PLA composite films (**h**).

Mechanical property is one of the most important properties of PLA composite films for further application. The mechanical performance of the PLA composite films was investigated, and we also used models to explain the results of the obtained film for analyzing the effects of the morphologies and dispersions of NCF on the tensile performance of the resultant composite films. The mechanical properties of neat PLA film and PLA composite films are shown in Figure 3a–g. The Elastic modulus (*E*), Tensile strength (*σ*), and Elongation at break (*ε*) values of neat PLA film were about 1.58 GPa, 32.26 MPa, and 3.6%, respectively. Compared with neat PLA film, the *E*, *σ,* and *ε* values of PLA/5CNF (5% CNF content) increased by 73%, 100%, and 26%, respectively (Figure 3a,d). This could be mainly attributed to the strong interface interaction (i.e., hydrogen bonding) between CNF and PLA matrix. In addition, the large aspect ratio of CNF (120–300) also contributed to the mechanical strength of PLA composites. Compared with the neat PLA film, 89%, 106%, and 0.28% improvements for *E*, *σ,* and *ε* were obtained for PLA/2CNC (2% CNC content), respectively (Figure 3b,e). It was probably due to the increased *CrI* and interface interactions between PLA and fillers. However, the higher crystalline index and rigid structure of CNC made the composite films more brittle. Moreover, as presented in Figure 3c,f, the *E*, *σ,* and *ε* values for PLA/5CF (5% regenerated CF content) increased by 72%, 59%, and 50%, respectively. From above, the composites incorporated with CNF with the larger aspect ratio (120–300) and the CNC with a higher rigid structure (*CrI*~74.7%) showed the stronger reinforcing effect on the mechanical property, as compared to the neat PLA film and the one with CF.

Compared with the neat PLA film, the mechanical properties of the PLA composite films increased progressively by adding the different types of NCF obtained in this work (Figure 3g). Figure 3h is the schematic illustration for the dispersion and fractured mechanism of PLA and PLA composite films. Additionally, the fractured surfaces of the PLA film and PLA composite films were investigated by SEM (Figure S1). It was found that the PLA film showed a relatively smooth and regular fractured surface (Figure S1), which attributed to the brittle nature of PLA [22]. Compared with neat PLA film, the PLA composite films exhibited a rougher surface. The nonuniform fractured surface suggested significant matrix deformation occurred, which benefited the improved mechanical properties of the prepared films. As shown in Figure S1b–d, abundant endpoints of NCF could be observed without visible microscale aggregation, and no significant differences in nanofiller dispersion were observed among the three samples, indicating the good interfacial compatibility between NCF and PLA matrix [22]. As shown in Figure 3h, due to the large aspect ratio and strong interface interaction, these nanofibers could also provide bridging effects for crazing, which also led to the enhancement of tensile performances. 

### 3.3. Roughness and Contact Angle Analysis

**Figure 4:**
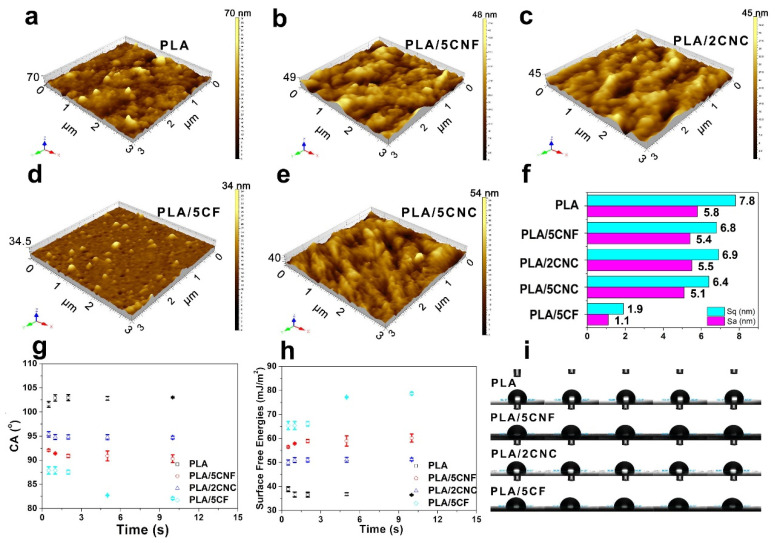
Three-dimensional, plotted images of neat PLA film (**a**), PLA/5CNF (**b**), PLA/2CNC (**c**), PLA/5CF (**d**), PLA/5CNC (**e**), respectively; Roughness (Sq) and average height (Ra) (**f**), water contact angle (**g**), surface free energies (**h**), and digital photo (**i**) of PLA film and PLA composite films.

It was reported that good interfacial interaction and filler dispersion were the premises for the final mechanical properties of composites [33]. Three-dimensional, plotted images were used to investigate the difference of the surface topography of the film samples (Figure 4a–e), and contour observation images are presented in Figure S2. The neat PLA film without NCF showed a relatively rougher surface compared to the PLA composite films. The rough surface of PLA was caused by the chain entanglement and agglomeration when the solvent was evaporated [34]. To better evaluate the surface roughness of the PLA and PLA composite films, roughness (*Sq*) and average height (*Sa*) were calculated from the AFM images (Figure 4f). It was found that *Sq* and *Sa* decreased with the incorporation of NCF. The difference of *Sq* and *Sa* between the PLA film and PLA composite films was mainly due to the following reasons. Firstly, for the neat PLA film, the solvent evaporation during drying could cause the entanglement of PLA chains [34,35]. Considering the fact that the evaporation rates of solvent at different evaporation points were irregular, this could cause the difference in the chain density of PLA, and, thus, the relatively rougher surface of neat PLA film was generated. However, the incorporation of well-dispersed NCF in PLA reduced the difference in the density, which was caused by the decrease of the chain mobility. Secondly, the ester groups of NCF with higher compatibility could have a considerable interaction with PLA matrix [12]. Therefore, the addition of NCF can make the surface of the composite films smoother compared to the neat PLA film. 

The ability of a surface to be wetted by water can be classified by its wetting contact angle (θ) of hydrophilic (θ < 90°), hydrophobic (90° < θ < 150°), or super-hydrophobic (θ > 150°) [36]. Figure 4g exhibits the change of water contact angle of the PLA film and PLA composite films in different contact time, and the digital photos are presented in Figure 4i. The surface of PLA/5CF was hydrophilic (θ = 87°), while the surfaces of the neat PLA film and the other PLA composite films were hydrophobic. With the introduction of NCF, the water contact angle of the PLA composite films was decreased, which was probably due to the incorporation of NCF with hydrophilic hydroxy. Moreover, the increase of *Sq* could slightly increase the water contact angle of the PLA composite films. The Fowkes model (Section S1.2, Supporting Information) was used to calculate the surface free energy, and the results are shown in Figure 4h [37,38]. The higher surface free energy indicated that the surface could be wetted easily (a small value of contact angle), which was in good agreement with the change of water contact angle. 

### 3.4. Thermal Stability 

**Figure 5:**
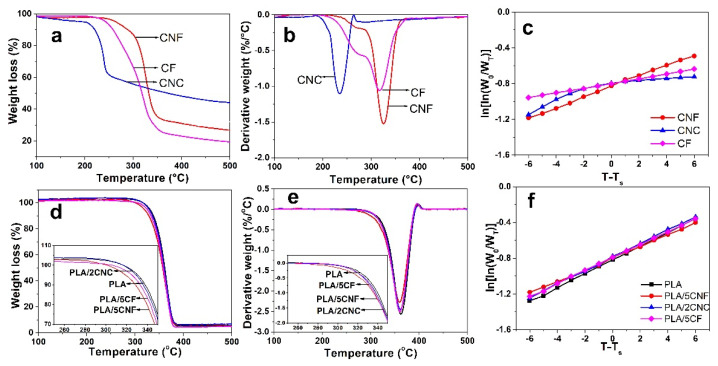
TG (**a**,**d**) and DTG (**b**,**e**) curves and plots of ln[ln(W_0_/W_T_)] vs. T–T_s_ (**c**,**f**) of NCF, PLA film and PLA composite films.

The thermogravimetric (TG) and derivative thermogravimetric (DTG) analyses were used to investigate the thermal behavior of NCF samples. Figure 5a,b shows the TG and DTG curves of NCF at a heating rate of 10 °C/min. It was reported that the initial slight weight loss below 100 °C was caused by vaporization and removal of the water imbibed in the samples [39]. The temperature region ranging from 220 to 400 °C was due to the thermal degradation of hemicellulose and cellulose [40,41,42]. Figure 5b shows that the thermal degradation of CNC was produced by a one-step process, indicating one type of uniform crystals with a narrow size distribution, while CNF and CF were degraded by a two-step process, and this behavior was probably due to the existence of an amorphous zone. Amorphous regions of cellulose are easier to be degraded compared to crystalline regions with an ordered and tight structure. The onset degradation temperature (*T_o_*) and maximum degradation temperature (*T_max_*) of raw materials and NCF are shown in Table 1. The CNC presented the lowest thermal stability compared with other NCF samples, which was probably due to the small size and big specific surface area [43]. Generally, the poor thermal stability of NCF will limit their application in thermally stable materials. Thus, the thermal stability of PLA composite films was further investigated. Figure 5d,e shows the TG and DTG curves of neat PLA film and PLA composite films. Compared with the neat PLA film, the thermal degradation curves of the PLA composite films exhibited a similar tendency of decomposition, which indicated that the thermal stability of PLA composites was relatively stable. The *T_o_* and *T_max_* of PLA film and composite films are shown in Table 1. Compared with PLA/CNC and PLA/CF, the PLA/CNF presented a lower *T_o_* value, which indicated the relatively lower thermal stability of PLA/CNF composites. This phenomenon was ascribed to the relatively lower *DS* (0.39) and large aspect ratio (120–300) of CNF [44,45]. The average apparent activation energies (*E_a_*, Section S1.3, Supporting Information) could be calculated from the plots of ln[ln(W_0_/W_T_)] vs. T–T_s_ (Figure 5c,f), and the results of *E_a_* are listed in Table S1. It was reported that a lower *Ea* represented a lower thermal stability, which was associated with lower *T_o_* values [46,47]. Compared to other NCF samples, CNF had larger *Ea* values, which was probably due to the relatively larger aspect ratio (120–300) and *CrI* (62.3%). Moreover, the *E_a_* values of PLA/CNF composite films were lower than those of other composite films, meaning that *T_o_* of PLA/CNF composites was relatively smaller, thus leading to a relatively lower thermal stability compared to other composite films.

### 3.5. Nonisothermal Crystallization Behavior and Crystallization Kinetics

**Figure 6:**
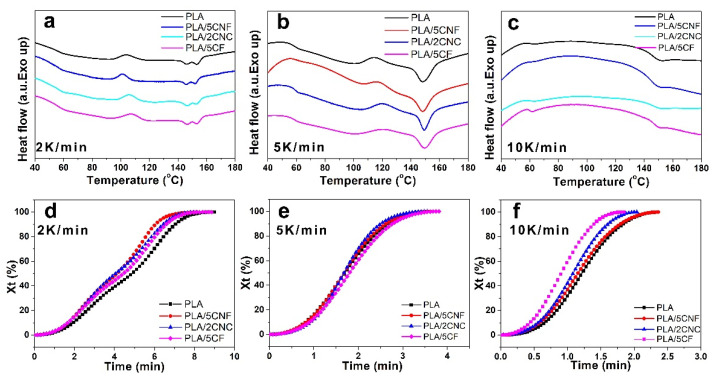
DSC curves of PLA film and composite films at different heating rates: 2 K/min (**a**), 5 K/min (**b**), and 10 K/min (**c**); Elative crystallinity curves for PLA film and composite films at different rates: 2 K/min (**d**), 5 K/min (**e**), and 10 K/min (**f**), respectively.

It is necessary to investigate the crystallization behavior of PLA, since the morphological, mechanical, and physical properties of PLA are controlled by its crystallization behavior [48]. Figure 6 shows the nonisothermal cold-crystallization DSC curves for PLA film and composite films in the temperature range from room temperature to 200 °C at different heating rates of 2, 5, and 10 K/min, respectively. At a slow heating rate (2 or 5 K/min), the molecular chains of PLA had enough time to self-regulate, leading to the strong cold-crystallization peak and melting behavior, which indicated the formation of a different crystal structure (Figure 6a,b). When the heating rate increased to 10 K/min, the endothermic and exothermic peaks were too weak to be observed. At this fast-heating rate (10 K/min), the PLA chains did not have enough time to self-regulate, leading to the weakened cold-crystallization peak (Figure 6c). Moreover, the presence of different multiple melting peaks indicated that the incorporation of different NCF led to the formation of a different crystal structure of the composite film. In order to investigate the effect of incorporation of three kinds of NCF samples, the crystallization kinetics were investigated. Figure 6d–f presents the relative crystallinity curves of PLA film and composite films, and the time of the extent of crystallization up to 50% (*t*_1/2_) is listed at Table S2. Clearly, the *t*_1/2_ values of composite films were lower than that of the neat PLA film, indicating that the crystallization rate of composite films became fast with the addition of NCF. The incorporation of NCF could increase the crystallinity rate, and NCF could also act as nucleation agents to form a more ordered crystalline structure and PLA crystal, which was more obvious at the low heating rate (2 K/min). Furthermore, the Avrami equation (Section S1.4, Supporting Information) was used to analyze the effect of NCF on the nucleation and crystallization rate of PLA film, and the results are presented in Figure S3 and Table S2. With the increase of the heating rate, a lower *k* value and *t*_1/2_ value were obtained, which indicated a faster crystallization rate. It should be mentioned that the *t*_1/2_ value of PLA/CF was lower than that of the other composite film and PLA film at 10 K/min, suggesting that the incorporation of well-dispersed CF with higher *DS* (>1.3) could improve the crystallization rate of PLA. Furthermore, the curves of log[−ln(1−X_t_)] vs. time had a good linear fitting degree, suggesting that this equation was suitable to describe the nonisothermal crystallization behavior of composite films (Figure S3). Thus, the presence of NCF could act as efficient nucleation agents to improve the crystallization rate of PLA, further proving the results of DSC analysis (Figure 6a–c).

### 3.6. Overall Migration Levels and Degradation Study

**Figure 7:**
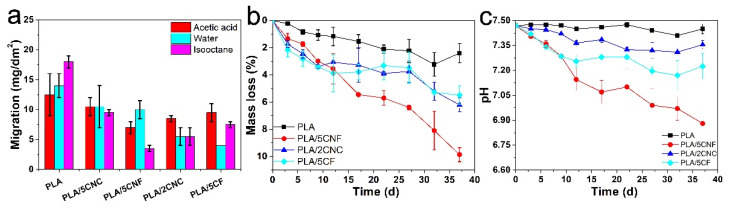
Overall migration levels (**a**), mass loss (**b**), and the change of pH (**c**) of PLA film and composite films.

It was reported that microplastics are potentially migrating from packaging materials into food; for this reason, simulants are most commonly used to test the overall migration levels of packaging polymers [49]. Figure 7a shows the overall migration levels of PLA film and composite films in three foods simulates (acetic acid, water, and isooctane). As reported, the overall migration limit for food contact material should be lower than 10 mg/dm^2^ [50,51,52]. The migration values of neat PLA film in isooctane were 18 mg/dm^2^. However, after incorporation with fillers, the migration of the composite films was reduced by 80.6%, 47.2%, 58.3%, and 69.4% for PLA/5CNF, PLA/5CNC, PLA/5CF, and PLA/2CNC, respectively. It should be mentioned that the migration of neat PLA/CNF in isooctane was lower than that of other composite films, which was probably due to the entanglements of CNF-restricted swelling of the biopolymer [53]. Moreover, the migration level of PLA/CF in water was slightly decreased, probably due to the good interface interaction between CF and PLA matrix. Furthermore, the mass loss (Figure 7b) and the change of pH (Figure 7c) were measured to investigate the degradation property of composite films. Cleary, the mass loss and the change of pH of composite films were higher than those of the PLA film, which was due to the hydrolysis and formation of low-molecule oligomers for PLA. In alkaline medium, the observed degradation may be explained by the cleavage of ester bonds and hydrolytic degradation of PLA, which generated more lactic acid [54,55,56]. It should be noted that PLA/CNF exhibited the best degradation property, which was probably due to the entangled network structure of CNF with a higher aspect ratio (120–300). Moreover, NCF with abundant hydroxyls groups could increase the contact surface of solutions and PLA matrix, which may be attributed to the degradation of PLA. Thus, composite films exhibited a faster degradation rate compared to that of neat PLA film.

## 4. Conclusions

In summary, three kinds of NCF with different amounts of surface ester groups were fabricated by one-step FA hydrolysis, and the impact of different properties (morphologies, *CrI,* and *DS*) of NCF on the quality of PLA composite films was systematically investigated. The incorporation of NCF in PLA could greatly improve the mechanical strength, crystallization ability, and degradation property of the composite films. The PLA/CNC film exhibited the highest increase (106%) of tensile strength, which was due to the incorporation of rod-like CNC with a higher *CrI*. PLA/CNF showed the best degradation property compared to CNC- and CF-based films, which was probably due to the entangled network structure of CNF with a higher aspect ratio (120–300). PLA/CF film showed the largest increase (50%) of elongation at break and more even surface, which was due to the stronger interfacial interaction between PLA and CF with higher *DS*. In addition, with the incorporation of NCF, the hydrophilicity and overall migration levels of the composite films relatively decreased, while the crystallization rate of the composite films increased. This fundamental study confirmed that the properties of NCF had a big impact on the end quality of the final PLA composite films, and the newly developed regenerated CF could be used as a nanofiller to reinforce PLA. Therefore, this work was very beneficial to reasonably choose NCF for the development of high-quality, sustainable packaging as an alternative to petroleum-based products.

## Figures and Tables

**Table 1:** Thermogravimetric data of NC, PLA film, and composite films.

	CNF	CNC	CF	PLA	PLA/5CNF	PLA/2CNC	PLA/5CF
*T_o_*/°C	309	228	279	344	336	341	341
*T_max_*/°C	325	244	310	365	360	362	360

## Supplementary Materials

The following are available online at https://www.mdpi.com/article/10.3390/nano12010001/s1. Experimental section, Figure S1: Cross-section images of neat PLA film (a), PLA/5CNF(b), PLA/2CNC(c), and PLA/5CF(d) films. Figure S2: Surface topography and Contour observation images of PLA films (a,b), PLA/5CNF (c,d), PLA/2CNC (e,f), PLA/5CF (g,h), and PLA/5CNC (i,j), respectively. Figure S3: Avrami equation parameters of relative crystallinity for PLA film (a), PLA/5CNF (b), PLA/2CNC (c), and PLA/5CF (d) composite films. Table S1: Thermal parameters of TS, NC, and composite films. Table S2: Avrami kinetic parameters of non-isothermal crystallization for neat PLA and composites.
