# Supplementary material for: Impact of the Incorporation of Nano-Sized Cellulose Formate on the End Quality of Polylactic Acid Composite Film
Source: Nanomaterials (Basel). 2021 Dec 21;12(1):1. doi: 10.3390/nano12010001 (PMC8746450; doi:10.3390/nano12010001)
Supplement: Supplementary file 1 [file nanomaterials-12-00001-s001.zip › nanomaterials-1519434-supplementary.pdf]

nanomaterials

MDPI

Supporting Information

Impact of the Incorporation of Nano-Sized Cellulose Formate on the End Quality of Polylactic Acid Composite Film

Yidong Zhang [1,2], Chao Liu [1], Meiyan Wu [1], Zhenqiu Li [2] and Bin Li [1,*]

1 CAS Key Laboratory of Biofuels, Qingdao Institute of Bioenergy and Bioprocess Technology, Chinese Academy of Sciences, Qingdao 266101, China; zhangyidongqust@163.com (Y.Z.); liuchao@qibebt.ac.cn (C.L.); wumy@qibebt.ac.cn (M.W.)
2 College of Marine Science and Biological Engineering, Qingdao University of Science and Technology, Qingdao 266011, China; lizhenqiu@qust.edu.cn
* Correspondence: libin@qibebt.ac.cn; Tel.: +86-532-80662725

S1. Experimental Section

S1.1. Degree of Substitution (DS) of NCF Samples

The *DS* values of NCF samples was determined according to the reported methods [1,2]. Briefly, the freeze-dried NCF sample (0.3 g) was mixed with 0.2 M potassium hydroxide in 50% aqueous alcohol (30 mL) in a 100 mL Erlenmeyer flask. Then, the flask was shaken at 100 rpm at room temperature for 24 h. After that, 0.2 M hydrochloric acid (30 mL) was added. A further half an hour later, the solution was titrated with 0.1 M sodium hydroxide, and phenolphthalein was used as an indicator. The formyl content of NCF sample was calculated by the formula (1), and then the *DS* of NCF sample was calculated by formula (2). Native as prepared bleached sample was also carried out using the same procedures as a blank.

$$Formyl\ (\%) = \frac{(V_S - V_B) \times N_{NaOH} \times M_{formyl} \times 10^{-3} \times 100}{W} \quad (1)$$

$$DS = \frac{162 \times Formyl\%}{M_{formyl} \times 100 - ((M_{formyl} - 1) \times Foryml\%)} \quad (2)$$

where, V_B (mL) is the volume of NaOH required for titration of the blank, V_S (mL) is the volume of NaOH required to titrate the NCF sample, N_{NaOH} is the normality of the NaOH solution, M_{formyl} is the molecular weight of the formyl group (29 g/mol), W (g) is the mass of sample used, and 162 is the molecular weight of the anhydroglucose units.

S1.2. Surface free energy of PLA film and PLA composite films

Fowkes model can be used to calculate surface free energy of film samples, and the equation of Fowkes model was as follows [3, 4]:

$$\gamma_S^d = \left(\frac{1}{4\gamma_L^d}\right)[\gamma_L^2(1 + cos\theta)^2] \quad (3)$$

where, subscripts S and L are the solid and liquid, γ_S^d is the surface free energy, θ is the contact angel, and the value of γ_L^d and γ_L were 72.8 mJ/m^2 and 21.8 mJ/m^2, respectively [5].

S1.3. Thermal stability

The average apparent activation energies (E_a) of thermal decomposition of NCF and film samples can be calculated by

$$ln[\,ln(\frac{W_0}{W_T})] = \frac{E_a\theta}{RT_S^2} \quad (4)$$

where, Ea is the average apparent activation energies, W_0 is the initial weight of the polymer, W_T is the residual weight of polymer at temperature T. T_S is the temperature determined at 36.79% of weight loss, θ is T-T_S, and R is the gas constant (8.314 J/(mol·K)).

S1.4. Nonisothermal crystallization behavior and crystallization kinetics

In order to study the effect of the NCF fillers on the crystallization kinetics of PLA composite films, the Avrami equation was used to describe the nonisothermal crystallization [2]:

$$X_t = \frac{\int_0^t (dH/dt)dt}{\int_0^\infty (dH/dt)dt} \quad (5)$$

$$lg[-ln(1 - X_t)] = lgk + nlgt \quad (6)$$

$$t_{1/2} = \left(\frac{ln2}{k}\right)^{1/n} \quad (7)$$

where, X_t is the relative crystallinity; *0, t,* and ∞ represent the relative crystallinity at time = *0, t,* and ∞, respectively; n is the Avrami exponent calculated from the slope of the liner fitting; k is the overall kinetic determined by intercept of the liner fitting; $t_{1/2}$ is the half-crystallization time.

S1.5. Overall migration tests

According to EU Directive 82/711EEC, the three simulants are water (simulant A) for aqueous foods; 3% acetic acid in water (simulant B) for acidic aqueous foods; and Isooctane (simulant C) for fatty foods, respectively [6]. The films with a total surface area of 10 cm^{-1} were immersed in a centrifuge tube with 10 mL for each simulant. After the reaction time (2 days) was over, the films were removed, the residue weight of simulants were determined. Then, overall migration of simulants was taken as the weight ratio of residue per total simulant [7].

S1.6. Degradation study

In order to study the degradation of composites, hydrolytic degradation at 37 °C was carried out in Phosphate Buffered Saline solution (PBS, mixing 0.27 g KH_2PO_4, 1.42 g Na_2HPO_4, 8 g NaCl and 0.2 g KCl in per 1000mL deionized water) [8]. The samples with surface area of 10 cm^{-1} were immersed in 10 ml PBS. The degradation of composites was characterized by measuring mass loss and change of pH [9].

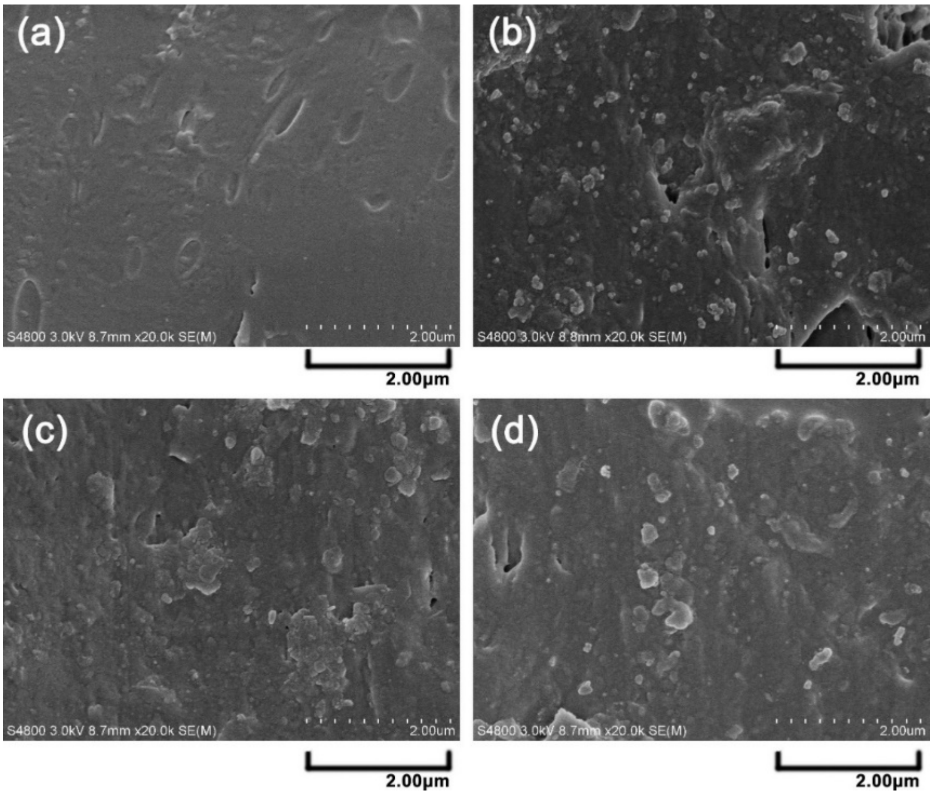

Figure S1. Cross-section images of neat PLA film (a), PLA/5CNF(b), PLA/2CNC(c), and PLA/5CF(d) films.

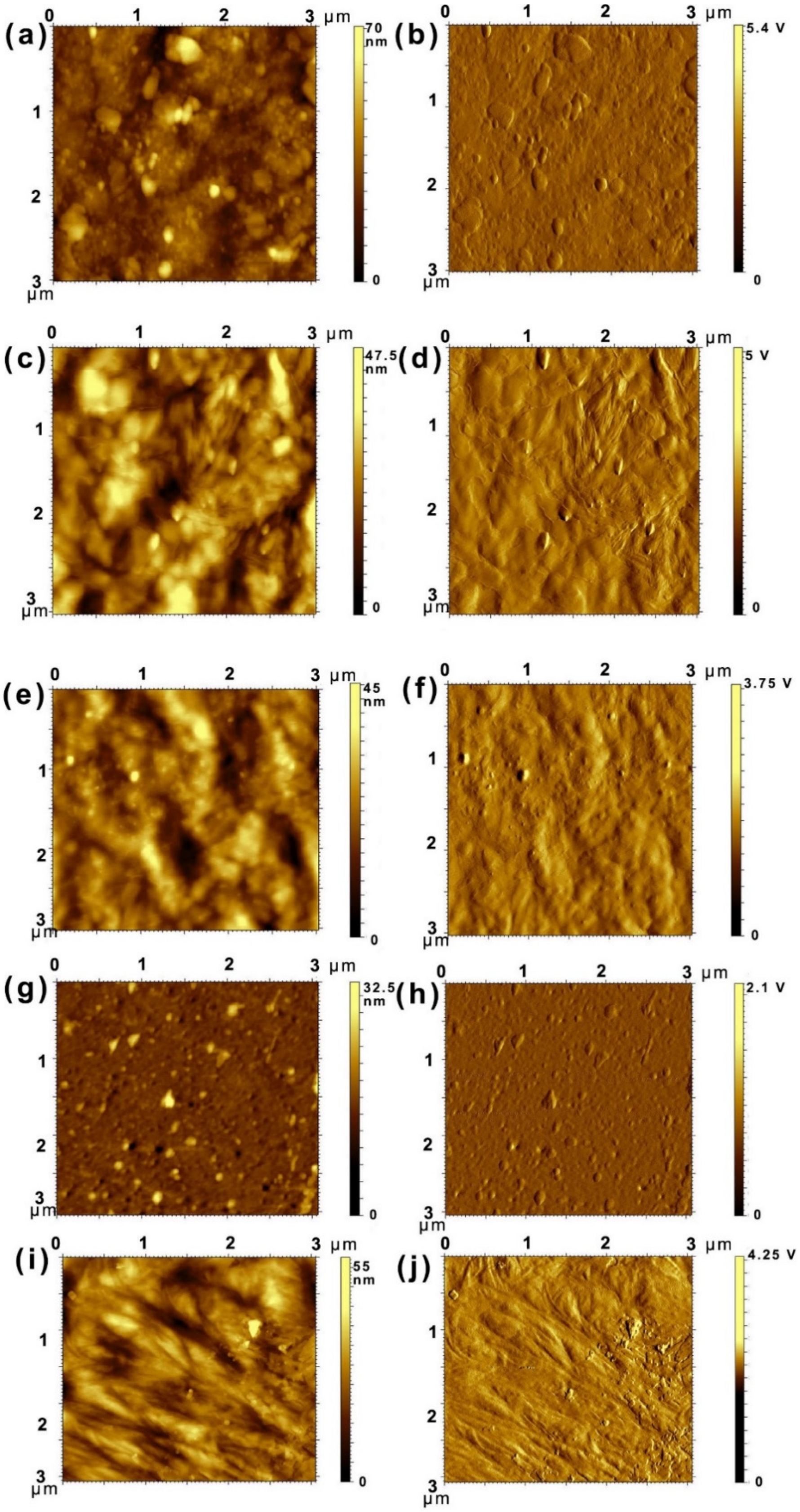

Figure S2. Surface topography and Contour observation images of PLA films (**a**,**b**), PLA/5CNF (**c**,**d**), PLA/2CNC (**e**,**f**), PLA/5CF (**g**,**h**) and PLA/5CNC (**i**,**j**), respectively.

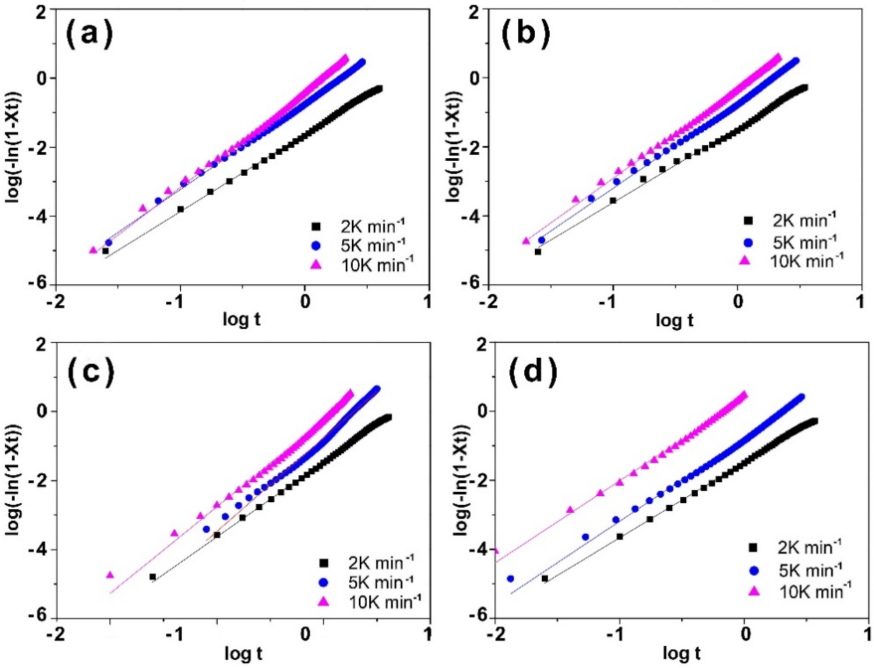

Figure S3. Avrami equation parameters of relative crystallinity for PLA film(**a**), PLA/5CNF(**b**), PLA/2CNC(**c**) and PLA/5CF(**d**) composite films.

Table S1. Thermal parameters of TS, NC, and composite films.

Sample	T_s (°C)	*Ea* (kJ/mol)
**TS**	344.90	21.44
**CNF**	340.90	49.23
**CNC**	244.10	9.77
**CF**	338.40	18.83
**PLA**	377.10	74.34
**PLA/5CNF**	375.30	64.05
**PLA/2CNC**	376.30	74.15
**PLA/5CF**	375.60	69.18

Note: TS is the temperature determined at 36.79% of weight loss of samples. *E*a is the average apparent activation energy of samples.

Table S2. Avrami kinetic parameters of non-isothermal crystallization for pure PLA and composites.

Sample	Heating rate (k▪min^{-1})	X_c (%)	n	k (min^{-n})	$t_{1/2}$ (min)
	2	4.7	2.24	2.34×10^{-2}	4.54
**PLA**	5	15.6	2.49	1.82×10^{-1}	1.71
	10	/	2.76	3.89×10^{-1}	1.23
	2	2.3	2.17	3.55×10^{-2}	3.93
**PLA/5CNF**	5	14.5	2.48	1.91×10^{-1}	1.68
	10	/	2.59	4.79×10^{-1}	1.15
	2	7.2	2.18	3.55×10^{-2}	3.91
**PLA/2CNC**	5	12.8	2.77	1.70×10^{-1}	1.66
	10	/	2.51	5.62×10^{-1}	1.09
	2	3.8	2.19	3.31×10^{-2}	4.01
**PLA/5CF**	5	15.5	2.40	1.66×10^{-1}	1.65
	10	/	2.38	2.34×10^{0}	0.60

References

1. H. Du; C. Liu; X. Mu; W. Gong; D. Lv; Y. Hong; C. Si; B. Li. Preparation and characterization of thermally stable cellulose nanocrystals via a sustainable approach of FeCl3-catalyzed formic acid hydrolysis. *Cellulose*. **2016,** 23(4), 2389-2407. http://doi.org/10.1007/s10570-016-0963-5.
2. H.Y. Yu; H. Zhang; M.L. Song; Y. Zhou; J. Yao; Q.Q. Ni. From Cellulose Nanospheres, Nanorods to Nanofibers: Various Aspect Ratio Induced Nucleation/Reinforcing Effects on Polylactic Acid for Robust-Barrier Food Packaging. *ACS applied materials & interfaces*. **2017,** 9(50), 43920-43938. http://doi.org/10.1021/acsami.7b09102.
3. M. Khodakarami; L. Alagha; D.J. Burnett. Probing Surface Characteristics of Rare Earth Minerals Using Contact Angle Measurements, Atomic Force Microscopy, and Inverse Gas Chromatography. *ACS Omega*. **2019,** 4(8), 13319-13329. http://doi.org/10.1021/acsomega.9b01491.
4. M.H. Gutierrez-Villarreal; F.J. Rodríguez-Gonzalez; Y. Perera-Mercado. Estimation of Surface Free Energy of Poly(lactic acid) During UV-Grafting with N-Vinylpyrrolidone. *Macromolecular Symposia*. **2017,** 374(1), 1600130. http://doi.org/10.1002/masy.201600130.
5. D.H. Kaelble. Dispersion-Polar Surface Tension Properties of Organic Solids. *The Journal of Adhesion*. **2008,** 2(2), 66-81. http://doi.org/10.1080/0021846708544582.
6. K. Grob. The future of simulants in compliance testing regarding the migration from food contact materials into food. *Food Control*. **2008,** 19(3), 263-268. http://doi.org/10.1016/j.foodcont.2007.04.001.
7. N. Burgos; I. Armentano; E. Fortunati; F. Dominici; F. Luzi; S. Fiori; F. Cristofaro; L. Visai; A. Jiménez; J.M. Kenny. Functional Properties of Plasticized Bio-Based Poly(Lactic Acid)_Poly(Hydroxybutyrate) (PLA_PHB) Films for Active Food Packaging. *Food and Bioprocess Technology*. **2017,** 10(4), 770-780. http://doi.org/10.1007/s11947-016-1846-3.
8. M. Vert; S. Li; H. Garreau. More about the degradation of LA/GA-derived matrices in aqueous media. *Journal of Controlled Release*. **1991,** 16(1-2), 15-26.
9. A. Naik; D.V. Shepherd; J.H. Shepherd; S.M. Best; R.E. Cameron. The effect of the type of HA on the degradation of PLGA/HA composites. *Materials science & engineering. C, Materials for biological applications*. **2017,** 70(Pt 1), 824-831. http://doi.org/10.1016/j.msec.2016.09.048.
